# Comprehensive study of algal blooms variation in Jiaozhou Bay based on google earth engine and deep learning

**DOI:** 10.1038/s41598-023-41138-w

**Published:** 2023-08-25

**Authors:** Bin Guan, Shaowei Ning, Xu Ding, Dawei Kang, Jiale Song, Hongwei Yuan

**Affiliations:** 1 School of Computer Science and Information Engineering, Hefei University of Technology, Hefei, 230009 China; 2 College of Civil Engineering, Hefei University of Technology, Hefei, 230009 China; 3 MOE Key Laboratory of Soft Soils and Geoenvironmental Engineering, Zhejiang University, Hangzhou, 310058 China; 4 Anhui & Huaihe River Institute of Hydraulic Research, Key Laboratory of Water Conservancy and Water Resources of Anhui Province, Hefei, 230088 China

**Keywords:** Ecology, Environmental sciences, Natural hazards

## Abstract

The Jiaozhou Bay ecosystem, a crucial marine ecosystem in China, has been plagued by frequent harmful algal blooms as due to deteriorating water quality and eutrophication. This study analyzed the temporal and spatial changes of harmful algal blooms in Jiaozhou Bay from 2000 to 2022 using the Floating Algae Index (FAI) calculated from MODIS (2000–2022) and Sentinel-2 (2015–2022) satellite image datasets. The calculation results of the image datasets were compared. The frequency of planktonic algal outbreaks was low and constant until 2017, but has increased annually since then. Algae blooms are most common in the summer and primarily concentrated along the bay’s coast, middle, and mouth, with obvious seasonal and spatial distribution characteristics. Several factors influencing algal outbreaks were identified, including sea surface temperature, wind speed, air pressure, dissolved oxygen, nitrogen and phosphorus ratios, chemical oxygen demand, and petroleum pollutants. Algal bloom outbreaks in Jiaozhou Bay are expected to remain high in 2023. The findings provide crucial information for water quality management and future algal outbreak prediction and prevention in Jiaozhou Bay.

## Introduction

The global proliferation of algal blooms has become a pressing environmental issue, primarily caused by environmental stressors such as eutrophication and oxygen depletion, which facilitate the excessive growth of phytoplankton or algae^1, 2^. These ecological issues not only disturb the equilibrium of aquatic ecosystems but also present a substantial peril to human health and the economy. The ramifications of algal bloom outbreaks can have significant implications, including the occurrence of fish mortality, the demise of aquatic vegetation, and the deterioration of water quality. The decomposition of algal blooms can lead to oxygen depletion and the discharge of perilous substances, exacerbating their adverse impacts on the aquatic ecosystem. Research indicates that optical observation techniques are effective instruments for investigating and monitoring Harmful Algal Blooms^3, 4^. Utilizing satellite imagery for monitoring planktonic algae has proven to be a valuable tool in improving our understanding of the mechanisms that cause algal outbreaks^5^. Furthermore, it has been demonstrated that ocean color indices derived from spectral band differences reliably provide information on algal outbreaks in both open and coastal waters^3^.

In recent years, Jiaozhou Bay, situated in the central Yellow Sea of China and on the southern coast of the Jiaodong Peninsula, has experienced frequent occurrences of green algae outbreaks. These outbreaks have had enduring impacts on the coastal carbon cycle and ecosystem^6–9^. In addition, the bay has become significantly impacted by industrial and domestic refuse discharges as a result of the expansion of economic activities and population. Therefore, nutrient concentrations in the bay have considerably increased, particularly from the Licun and Dagu rivers^10^. The high risk of harmful algal bloomsin Jiaozhou Bay, similar to the Gulf of Mexico, is mainly attributed to the bay’s rich nutrients and frequent water exchange with the Yellow Sea^11^.It has been found that the chlorophyll a concentration in the Jiaozhou Bay region is higher in summer, further corroborating the occurrence of algal blooms in the region^12^. The significant changes in the shoreline of Jiaozhou Bay and the reduction of the bay area and tidal prism have led to a decrease in hydrodynamics, hindering the diffusion of nutrients within the bay but accelerating their accumulation, thus contributing to the emergence of planktonic algal blooms^13^.

Earlier research on the community structure and species of planktonic algae in Jiaozhou Bay involved collecting samples and performing microscopic observations for classification and identification^14, 15^. However, remote sensing satellites can cover vast areas of seas and lakes, providing high spatial and temporal resolution data for timely detection and monitoring of algal bloom distribution, extent, and changes^16, 17^. The Floating Algae Index (FAI) has been shown to provide superior estimates of algal bloom coverage^13, 18^. Several studies are currently using optical remote sensing satellites and the FAI index to monitor algal bloom areas in lakes such as Taihu Lake, Dianchi Lake, and Chaohu Lake^19–22^. However, few studies have focused on the surveillance of planktonic algae in semi-enclosed bays like Jiaozhou Bay.

In this study, the spatial and temporal dynamics of algal blooms in the Jiaozhou Bay region from 2000 to 2022 were inverted using MODIS and Sentinel-2 satellite imagery. The study focuses on the following major issues: (1) The algal bloom extraction methods were constructed using the Google Earth Engine cloud platform (GEE) based on MODIS and Sentinel-2 datasets for the periods of 2000–2022 and 2015–2022, respectively. Algal bloom areas were extracted and the temporal and spatial changes of algal blooms in Jiaozhou Bay from 2000 to 2022 were analyzed. (2) The causes of the algal bloom in Jiaozhou Bay were investigated using meteorological and nutrient factors, as well as regression analysis. (3) Deep neural network (DNN) and Seasonal Autoregressive Integrated Moving Average (SARIMA) model prediction methods were used to model and predict the change of algal area in Jiaozhou Bay (Supplementary information).

## Methods

### Study area

**Figure 1:**
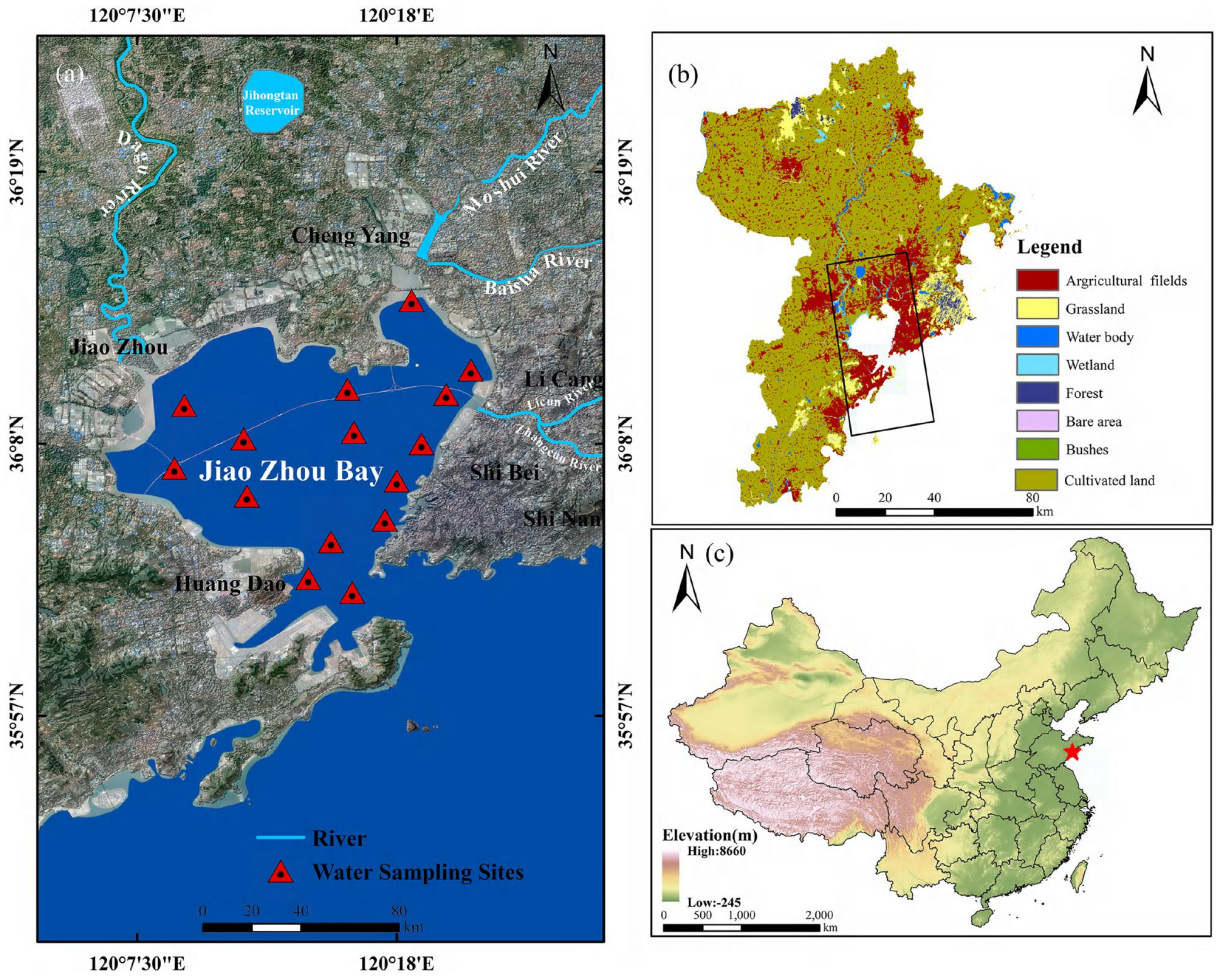
Study area information including distribution of observatories and rivers discharged into Jiaozhou Bay and landuse around Jiaozhou Bay. Map created with ESRl ArcGIS 10.8(https://www.esri.com/en-us/arcgis/products/index), backgroud map of (**a**) is Sentinel-2 image composite from 2021-04-01 to 2021-10-31 after de-cloud (https://developers.google.com/earth-engine/datasets/catalog/sentinel-2) and the review number of China Map is GS (2020) 4634. (**b**) Land use in Qingdao. The black box indicates the location of Jiaozhou Bay. (**c**) Location of Jiaozhou Bay in China.

Jiaozhou Bay ($120^\circ \;10^{\prime} - 120^\circ \;37^{\prime}{\text{E}},\;36^\circ \;06^{\prime} - 36^\circ \;25^{\prime}{\text{W}}$), located in the central Yellow Sea of China, is a semi-enclosed bay that covers the southern coast of the Jiaodong Peninsula in Qingdao, Shandong Province.The bay has a near-trumpet shape, with a maximum length of about 40 km in the north-south direction and a maximum width of about 28 km in the east-west direction. Its average depth is 7 m and its maximum depth is 64 m. With a total area of 438 km^2^, Jiaozhou Bay is the third-largest bay in China (Fig. 1). Due to its monsoon climate, the bay experiences the southeast monsoon in summer and the northwest monsoon in winter. Several small-scale circulation currents, such as a counter-current and a rotating current, are caused by the influence of the Yellow Sea Current on the bay.

Jiaozhou Bay is a typical eutrophic ecosystem, where the chlorophyll a concentration is highest in the bay’s north-eastern and north-western regions and progressively decreases southward. August marks the annual maximum and maximal fluctuations of chlorophyll a^23^. The total organic carbon (TOC), total nitrogen (TN), and carbon-to-nitrogen (C/N) ratios in the solid phase of Jiaozhou Bay sediments increase progressively, primarily as a result of the extensive pollution from human inputs via river discharges^24^. Concentrations of phytoplankton are greatest in the northwestern and northern regions of the bay, near the river headwaters, and decrease with increasing depth from the inner to the outer bay. Due to nutrient accumulation under south-eastern wind conditions, phytoplankton epidemics may occur near the northwestern coast^25^. Over the past three decades, Jiaozhou Bay has experienced a considerable increase in pollutants, resulting in a decline in water quality^26^.

### Data

#### MODIS data

The MOD09GA Version 6.1 product is a remote sensing data product collected by the Moderate Resolution Imaging Spectroradiometer (MODIS) sensor on board a satellite. It captures a single image of the Earth’s surface once per day with a spatial resolution of 500 meters.

In this study, we selected MOD09GA.061 Terra Surface Reflectance Daily Global 500-meter satellite data from January 2000 to November 2022 to examine the evolution of algal blooms in the Jiaozhou Bay region. We obtained a total of 8216 images through data selection in Google Earth Engine and used them as one of our data sources. The data were obtained from the Earth Engine Data Catalog platform(https://developers.google.com/earth-engine/datasets/catalog/MODIS_061_MOD09GA).

#### Sentinel-2 data

Sentinel-2 is a high-resolution multispectral imaging satellite equipped with a Multi-spectral Instrument (MSI). It comprises two identical satellites, Sentinel-2A and Sentinel-2B, which operate simultaneously^27^. The satellite has a spatial resolution of 10 meters and a temporal resolution of 5 days, with each satellite observing every 10 days and alternating. The data were obtained from the Earth Engine Data Catalog platform(https://developers.google.com/earth-engine/datasets/catalog/sentinel-2).

In this study, 460 scenes of Level-1C data from August 2015 to December 2021 and 246 scenes of Level-2A data from October 2018 to December 2021 were extracted after cloud filtering with Google Earth Engine for the study of algal blooms in the Jiaozhou Bay region.

#### Meteorological data

The meteorological data used in this investigation were collected from the Xiaomai Island Observatory and consisted of hourly observations of air temperature, sea surface temperature (SST), wind speed, wind direction, and air pressure from July 2010 to July 2022. These data were collected by the National Ocean Data Observation Centre (http://mds.nmdis.org.cn/). Monthly averages were calculated to investigate the meteorological factors that initiate harmful algal blooms in Jiaozhou Bay.

#### Water quality monitoring data

This study utilized measurements of dissolved inorganic nitrogen (DIN), dissolved inorganic phosphorus (DIP), chemical oxygen demand (COD), dissolved oxygen (DO), and petroleum pollutants for water quality monitoring. From 2014 to 2022, these measurements were obtained in the near-shore waters of Jiaozhou Bay. In addition, from 2014 to 2018, measurements of total nitrogen (TN), total phosphorus (TP), and silicate were collected from four inlet rivers in the Jiaozhou Bay area, including the Licun River, Dagu River, Moshui River, and Haibo River. The information was gathered at the National Field Scientific Observation and Research Station(http://jzb.cern.ac.cn/) for Marine Ecosystems in Jiaozhou Bay, Shandong Province, and all the sampling points of the station have been fully marked in Fig. 1a.

### Image processing methods

#### Floating algae index (FAI)

The Floating Algae Index (FAI) employs a threshold segmentation method that utilizes the red, near-infrared (NIR), and shortwave infrared (SWIR) bands to extract areas of algal blooms in seawater. This method emphasizes waters affected by algal blooms by using the uplift of algal blooms in the NIR band and reduces the FAI’s sensitivity to different aerosol types by subtracting the baseline^18^. The reduced susceptibility of FAI to environmental interference and its enhanced advantages over other methods, such as the Normalized Difference Vegetation Index (NDVI) and Enhanced Vegetation Index (EVI), have led to its increased use in the global study of algal blooms^28–31^.

1 $$\begin{aligned} {\text{FAI}} = & R_{{rc,NIR}} - R_{{rc,NIR^{\prime } }}^{\prime } \\ R_{{rc,NIR}}^{\prime } = & R_{{rc,{\text{ Red }}}} + \left( {R_{{rc,SWIR}} - R_{{rc,{\text{ Red }}}} } \right) \times \left( {\lambda _{{NIR}} - \lambda _{{{\text{ Red }}}} } \right)/\left( {\lambda _{{SWIR}} - \lambda _{{{\text{ Red }}}} } \right) \\ \end{aligned}$$

where $R_{{rc,NIR}}$,$R_{{rc,\text{Re} d}}$ and $R_{{rc,SWIR}}$ are the reflectance in the NIR, Red and SWIR bands after Rayleigh correction, respectively. $\lambda _{{{\text{ NIR }}}}$, $\lambda _{{{\text{Red}}}}$ and $\lambda _{{{\text{SWIR}}}}$ are the central bands of the sensor’s corresponding bandwidth.

#### Selection of the optimal threshold

To accurately extract the area of algal colonies using FAI threshold segmentation, it is necessary to identify the optimal threshold that yields the most accurate results. However, it is not possible to manually determine the optimal threshold through point-by-point observation and experimentation on a single image. Similarly, manually determining the optimal threshold for all MODIS and Sentinel-2 images and then performing statistical analysis to identify the values would be a daunting task. The maximum gradient method introduced by Ma et al.^21^ is an effective alternative to manually determining the optimal threshold. However, it should be noted that this method requires a substantial amount of human effort. In addition, it is essential to recognize that a large number of images without algal blooms will need to be analyzed and their optimal thresholds determined, which can be time-consuming and may affect the statistical regularity of the optimal thresholds for algal bloom outbreaks.

This research differs from others in that it concentrates on continuously observing the extraction effect of various thresholds on an image of an algal outbreak (captured on 10 July 2021) in order to determine the optimal threshold as the starting point for analysis. Despite the possibility that the starting threshold does not entirely reflect the optimal extraction effect, it can still reflect the trend of algal bloom area change. The study then traversed all images that met the initial threshold in order to isolate the algal blooms region. By choosing the larger FAI area as the date of the algal bloom, a threshold for determining the image set was established.

The study determined manually, with a minimum accuracy of 0.005, the optimal threshold value for each image in the set and analyzed the results. The most frequent threshold value was selected as the optimal threshold value, saving time and preventing the capture of images free of algal blooms. In the Jiaozhou Bay region, the optimal FAI extraction threshold was determined to be 0.05 for MODIS and 0.045 for Sentinel-2 using this method.

#### Processing of MODIS satellite data

In this study, we processed the MODIS satellite data using the Google Earth Engine cloud platform (GEE) (https://code.earthengine.google.com/)^32^. After reprojecting the MOD09GA dataset from ’SR-ORG:6974’ to ’EPSG:4326’, we clipped the images to our study area and kept the resolution constant at 500 meters. In order to investigate algal bloom outbreaks in the region, we additionally removed clouds from each image. During processing, we filtered the data and selected only those with more than zero observations. Then, these particular data were declouded. For this research, we selected a band resolution of 500 meters, which may introduce some error when analyzing small areas such as Jiaozhou Bay. To prevent erroneous positives caused by the involvement of the ground in the calculation process, a special treatment was applied to the study area. Specifically, we selected only data within 1 kilometer of the coast. Ultimately, the algal bloom zone was extracted.

#### Processing of Sentinel-2 satellite data

The GEE platform provides Sentinel-2 images in Level 1C and Level 2A formats. The first is orthorectified and geometrically corrected but lacks atmospheric correction, whereas the second is ortho-corrected for bottom-of-atmosphere reflectance (BOA). Consequently, Level 2A images provide more realistic reflectance data, more accurate color levels, greater brightness and contrast, and are generally more color sensitive. In the previous study, we found that atmospheric correction was predominantly responsible for the difference in quality. When extracting the area of algal blooms using the FAI threshold segmentation method, the area of Level 2A data was consistently greater than that of Level 1C data for bloom areas that exceeded a certain threshold.

**Figure 2:**
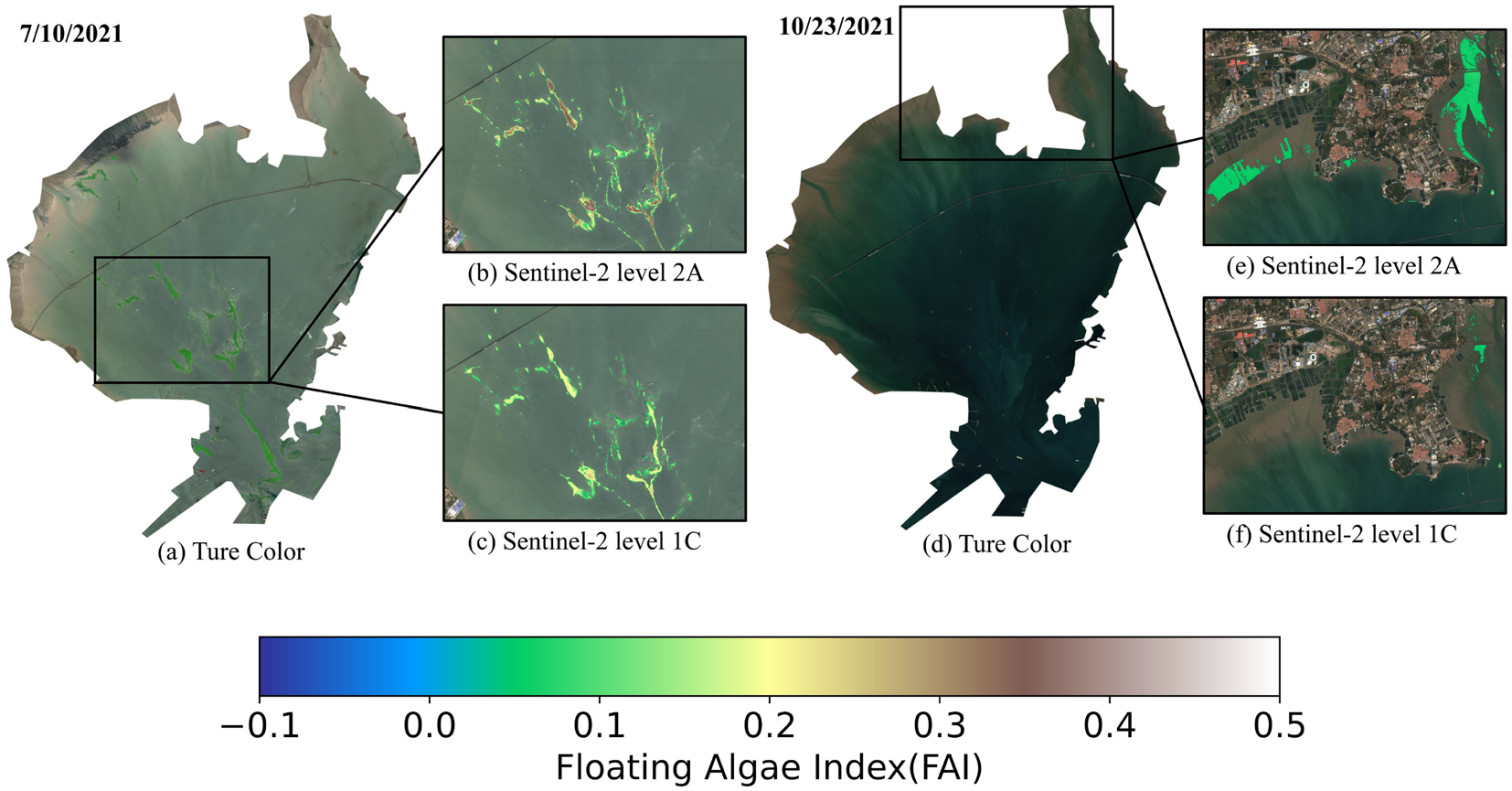
Comparison of FAI index extracted from Sentinel-2 2A and 1C at the same threshold The map was created by using Python 3.7(https://www.python.org/downloads/).

**Figure 3:**
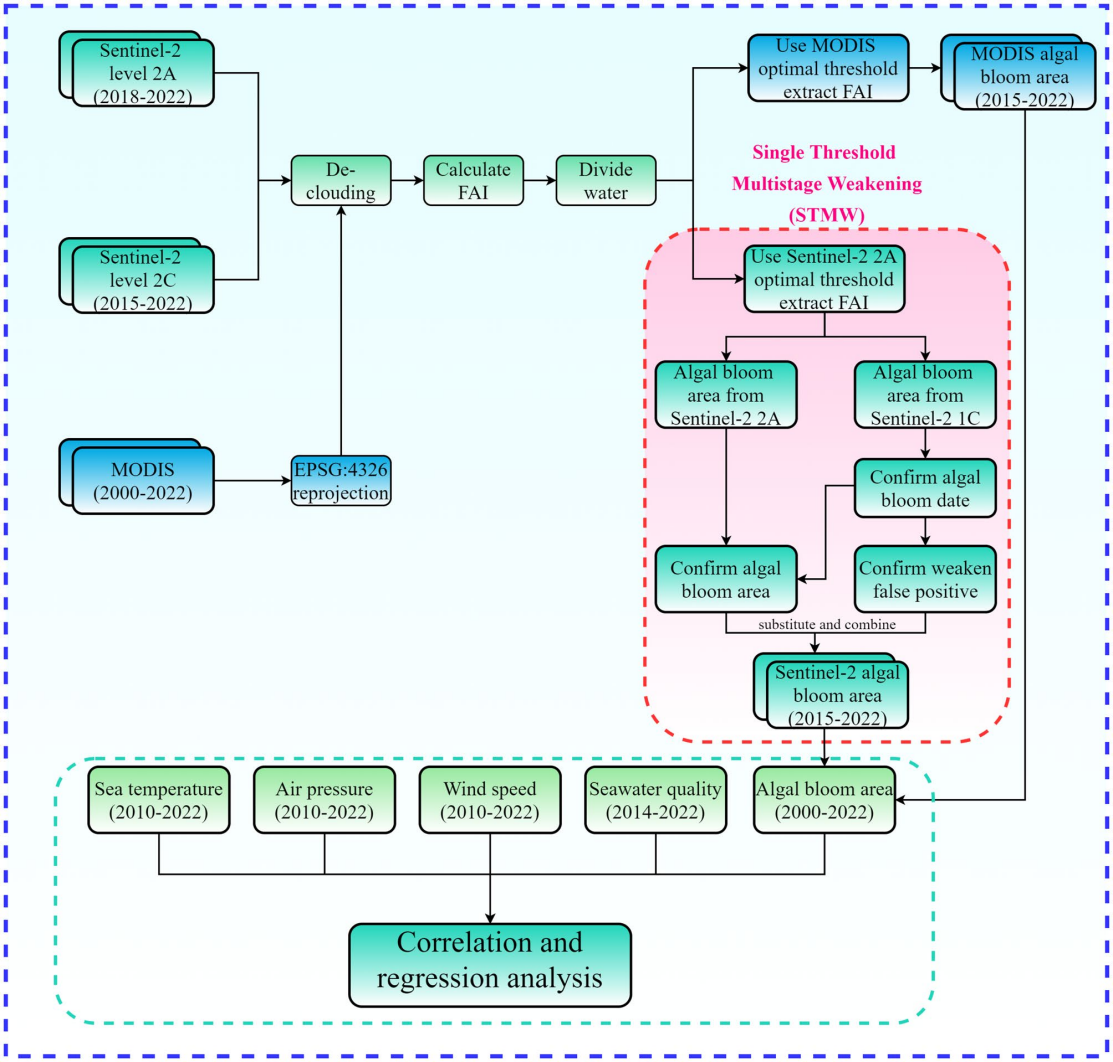
Flow chart for extracting the area of algal blooms using the GEE.

In this study, two images were extracted using the 1C-Level and 2A-Level datasets from Sentinel-2 (Figure 2) on 10 July 2021, an algal bloom outbreak was observed, and analysis of the extracted area and color depth revealed that the FAI value for dateset 2A was greater than for dataset 1C. Consequently, when both were extracted above a particular threshold, the outbreak area of dataset 2A was greater than that of dataset 1C. On October 23, 2021, no algal bloom outbreak was detected. Nevertheless, it was discovered that dataset 2A was more sensitive due to its own atmospheric correction procedure and Sentinel-2’s high resolution. This increased the number of false-positive planktonic algae identifications^33, 34^. In this study, the false-positive phenomenon occurs when non-algal bloom areas are extracted as algal blooms using satellite imagery. Due to factors such as the developed fisheries industry, human activity, and changes in sea level in the coastal waters of Jiaozhou Bay, shallow beaches and sediment often appear near the coast. These areas may be mistakenly extracted as algal blooms using the FAI method, resulting in false positives. In contrast, fewer false positives were extracted from the dataset 1C. Although the threshold can be adjusted to reduce false positives, this may result in algal bloom area loss, which may not be desirable^21^. This study proposes the Single Threshold Multi-stage Weakening (STMW) method (as shown in Figure 3) to reduce the occurrence of false positives while mitigating the loss of algal bloom area.

The STMW method was developed to overcome the limitations of using either level 1C or level 2A data by combining the two. The 1C data can be used to identify the days of algal blooms outbreak, but the actual aera of algal blooms outbreak is calculated from the corresponding 2A data. This method extracts the area of the blooms from a copy of the level 2A data at its optimal threshold and utilizes the level 1C data to substantially reduce the number of false positives in the level 2A data. The STMW method utilizes the low sensitivity of the 1C data to eliminate the effect of false positives and precisely analyze the number of algal bloom outbreak days. Finally, it eliminates some outliers and reduces errors, thereby enhancing the detection accuracy of algal bloom areas.

#### Modeling the area of algal bloom

##### Deep neural networks (DNNs)

 Predicting changes in the area of algal outbreaks remains challenging due to the heterogeneity of algal blooms and their unpredictable response to complex environmental conditions^35^. This study employs deep neural networks (DNNs), a type of deep learning technique, to analyze changes in the area of algal bloom outbreaks, focusing exclusively on the influence of meteorological factors. DNNs are generally accepted as effective in predicting time series data^36^.

**Figure 4:**
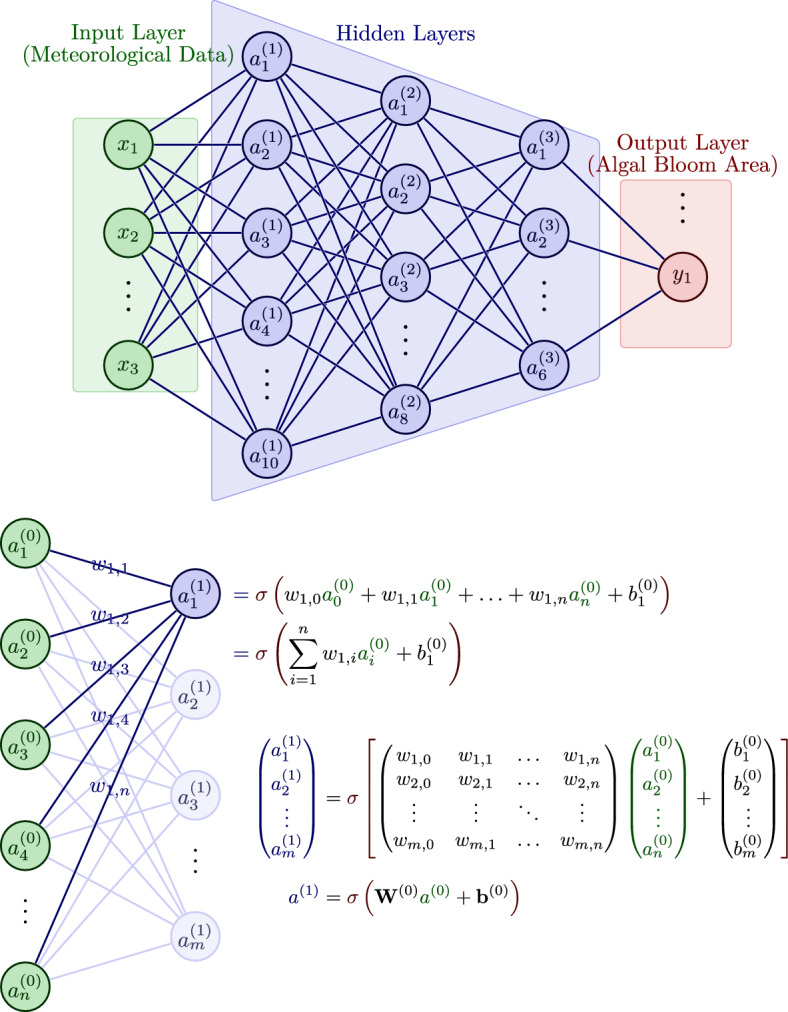
Structure diagram of deep neural network.

This study selected the area of algal blooms measured using MODIS since 2010 and calculated the monthly average of this data. The relationship between this area data and three meteorological factors - wind speed, barometric pressure, and sea surface temperature (SST) - was analyzed separately (Figure 11). The results showed that the changes in algal bloom area exhibited a distinct annual variation pattern and had a strong correlation with these three factors. In this paper, we used a DNN neural network model (Figure 4) to predict the monthly mean algal bloom area in the study area, with wind speed, barometric pressure, and SST as independent variables and algal bloom area as the dependent variable.

##### SARIMA model

Changes in the time series of algal bloom areas show that these outbreaks exhibit strong seasonality, clear cyclical characteristics, and growth trends. However, the previous deep neural network (DNN) model had limitations in accurately predicting algal outbreaks under the influence of multiple factors due to variability among indicators. Furthermore, because the input layer only used meteorological factors, the model lacked data interpretability. To improve prediction accuracy, we used seasonal decomposition and the Seasonal Autoregressive Integrated Moving Average (SARIMA) model to control the objective pattern of algal development. The SRIMA model is an extension of the ARIMA model, which was originally proposed by Box and Jenkins in 1970 for time series analysis^37^. The SRIMA model incorporates seasonal components by including seasonal differencing to capture seasonal variations in the time series data. By exclusively combining the 22-year extraction results of the MODIS dataset, we analyzed and predicted the algal bloom area in time series from an alternative perspective.

The MODIS extraction results revealed an upward trend in algal bloom outbreaks, with significantly stronger outbreaks occurring in the second and third quarters than in the first and fourth quarters. Despite changes over time, the seasonal fluctuations of the algal bloom area did not differ significantly between the previous and current observation periods. This study removes the influence of seasonality from the time series to investigate the seasonality of algal outbreaks and other masked characteristics. To accomplish this, we used an additive seasonal decomposition model to divide the algal bloom outbreak time series into four components: irregular changes, seasonally adjusted series, seasonal adjustment factors, and trend cyclic components. These elements enable us to quantify the effects of different factors on algal bloom outbreaks. After testing, we used SARIMA (0, 0, 1) (0, 1, 1) to analyze and predict based on the above patterns.

2 $$\left( {1 - L^{{12}} } \right)y_{t} = \alpha _{0} + (1 + \theta L)\left( {1 + \Theta L^{{12}} } \right)\varepsilon _{t}$$

where L is the lag operator and $\varepsilon _{t}$ is a white noise series with variance $\sigma ^{2}$. $\Theta$ is the seasonal moving average term coefficient, $\theta$ is the non-seasonal moving average term coefficient and $\alpha _{0}$ is the intercept term.

## Results

### Comparison of algal bloom observation areas using different satellites

**Figure 5:**
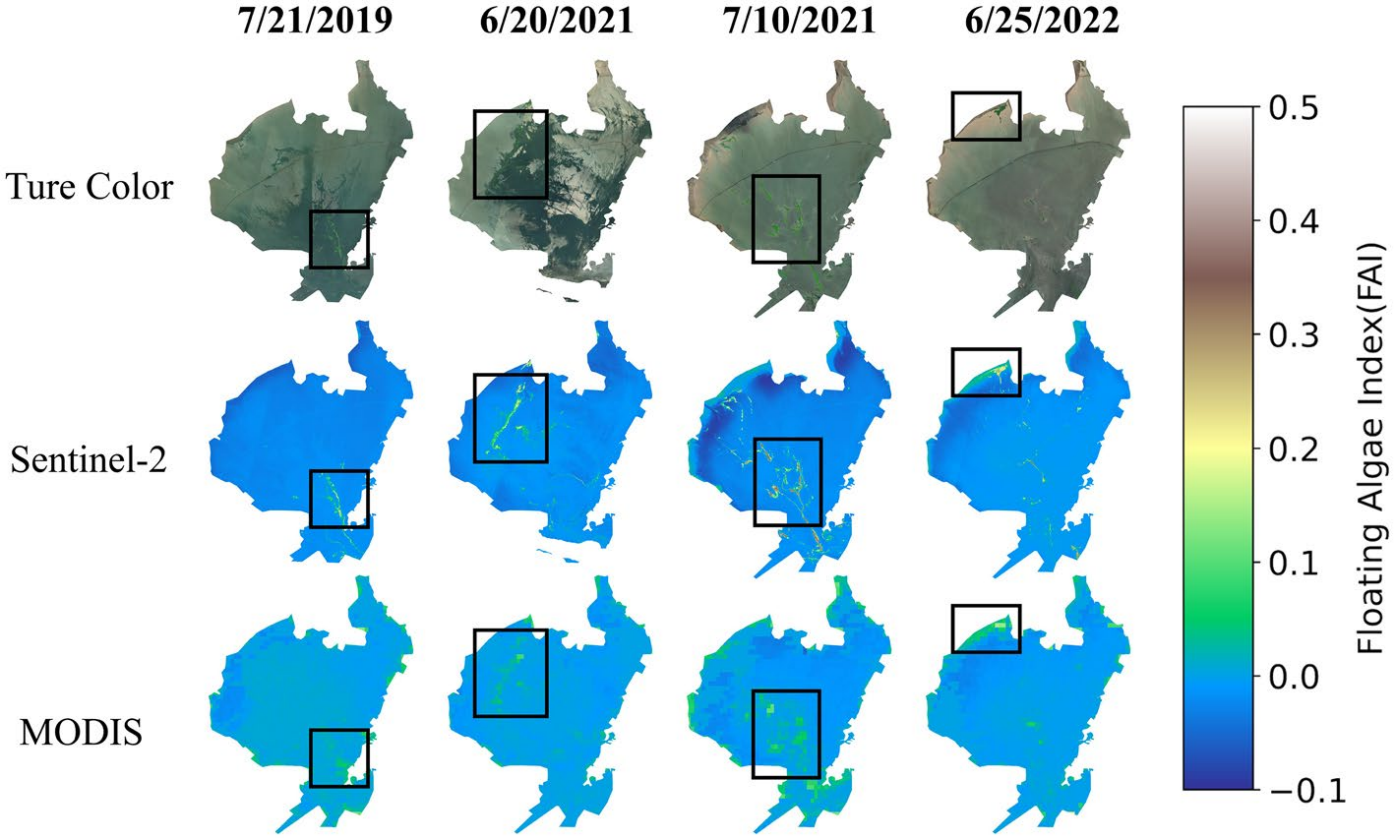
Example diagram of FAI extraction. First row is the Sentinel-2 true color image, Second and third row are the FAI calculated fromSentinel-2 and MODIS at the same day(21th July 2019, 20th June 2021, 10th July 2021, 25th June 2022).The map was created by using Python 3.7 (https://www.python.org/downloads/).

**Figure 6:**
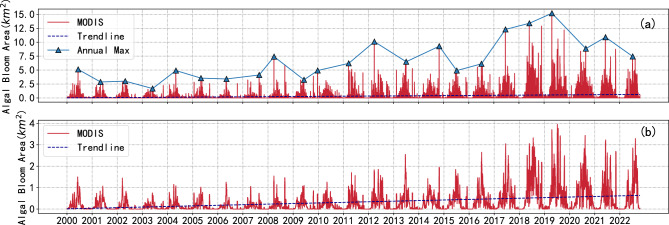
(**a**) Time series of algal blooms area in Jiaozhou Bay from 2000 to 2022. (**b**) Time series of algal blooms area processed by MA window in Jiaozhou Bay from 2000 to 2022.

**Figure 7:**
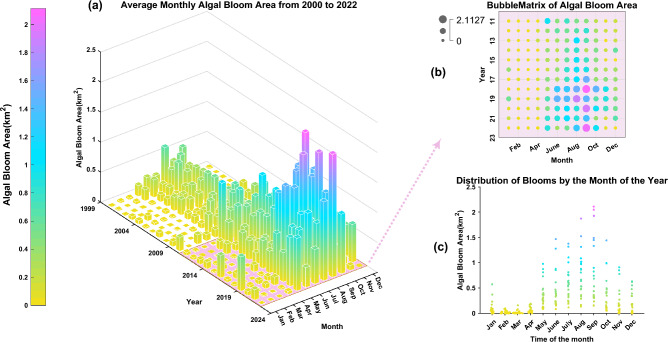
Monthly average algal bloom area from 2000 to 2022 (**a**), (**b**) shows the bubble matrix of algal bloom area from 2011 to 2022, (**c**) shows the distribution of algal blooms occurring in different months.

Multiple satellites were utilized to track the algal bloom in Jiaozhou Bay. For comparison, we selected three images from each of four days: the Sentinel-2 true-color image, the Sentinel-2 FAI index image, and the MODIS FAI index image.These images were compared to evaluate their ability to extract information about the algal bloom region (Figure 5).Both MODIS and Sentinel-2 images can detect algal bloom outbreaks, but they differ significantly in spatial resolution. MODIS has a lower spatial resolution, resulting in coarser monitoring results for cyanobacterial outbreak areas. However, its high temporal resolution allows for observation of the entire outbreak process. Using the extracted area from the MODIS dataset, we generated a 22-year time series of variations in the algal bloom area (Figures 6 and 7). To better analyze the trend of changes in the algal bloom area in Jiaozhou Bay, we used a moving average (MA) window method (Figure 6(b)). The MA window is a commonly used technique to smooth out the fluctuations in a time series and identify trends or patterns. The moving average is calculated by taking the average of a fixed number of consecutive data points in the time series. This fixed number is known as the window size. In this study, we set the window size as seven days. According to our findings, the area of algal blooms in Jiaozhou Bay remained relatively stable prior to 2017. However, over the next five years, there was a general upward trend in the overall algal bloom area in Jiaozhou Bay. Further examination of the annual changes in algal blooms reveals that algal bloom outbreaks are primarily concentrated in the summer, particularly from May to September. This finding is consistent with previous research on chlorophyll a concentrations in Jiaozhou Bay, which discovered that the peak concentration of chlorophyll a occurs during the summer months^12^.

**Figure 8:**
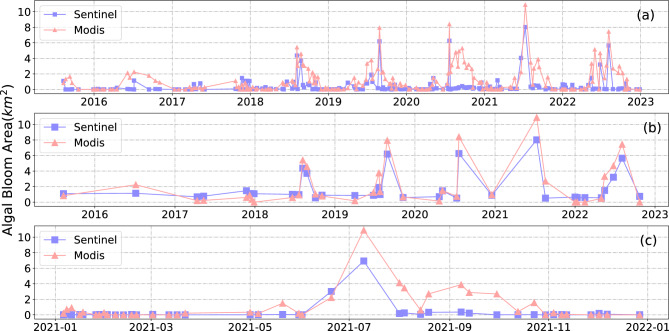
Comparison of FAI index calculated by MODIS and Sentinel-2. (**a**) is the result from the original data without any processing, (**b**) is the result that eliminated area less than 0.7${\text{km}}^{2}$. (**c**) is the result for 2021.

We compared the algal bloom area data extracted from Sentinel-2 and MODIS and examined the differences and effects of extraction between the two satellites (Figure 8). Due to the discontinuity of the Sentinel-2 time series during the extraction process, we selected corresponding MODIS values for comparison based on the availability of Sentinel-2 data. Both MODIS and Sentinel-2 successfully captured peak periods of high algal outbreaks, demonstrating their effectiveness in monitoring algal blooms. However, we observed some false positives in MODIS data during non-outbreak periods.

**Figure 9:**
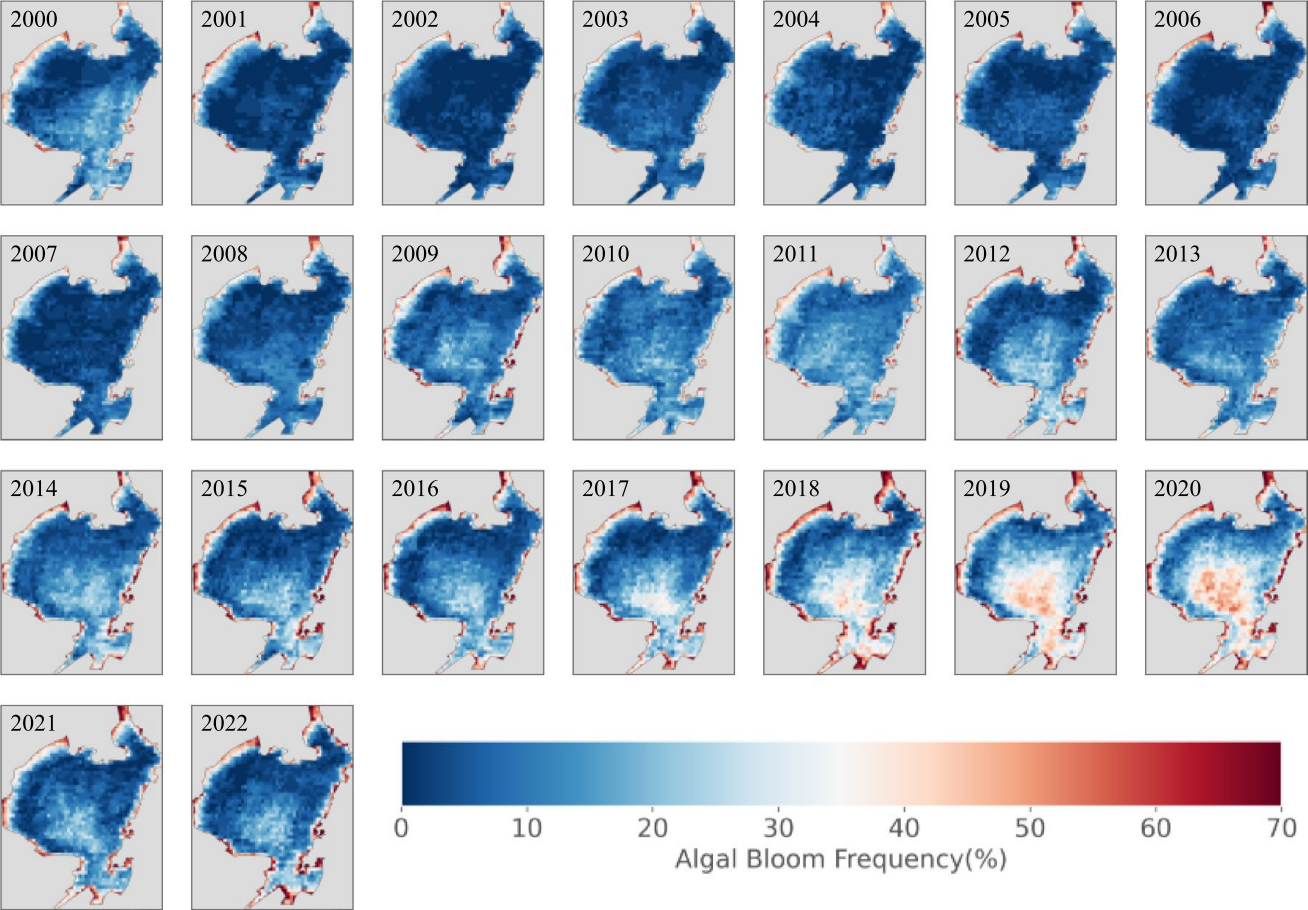
Spatial frequency distribution of the algal bloom area in Jiaozhou Bay from 2000 to 2022 extracted by MODIS .The map was created by using Python 3.7 (https://www.python.org/downloads/).

Despite these differences, there was strong consistency in the trends between the two datasets. Additionally, the value of the algal bloom area in the MODIS data was higher than that of Sentinel-2, which can be attributed to differences in resolution and sensors used by the two satellites. Figure 9 shows the frequency of spatial distribution characteristics of algal blooms in Jiaozhou Bay. Our findings indicate that the coastal area of Jiaozhou Bay experienced a more severe outbreak of algal blooms, with the area of algal blooms decreasing from the coast to the bay’s inner sea. The outbreak was primarily concentrated in the bay’s coastal, central, and estuary regions. We analyzed the data on total nitrogen, total phosphorus, and silicate content in the Li River, Da Gu River, Mo Shui River, and Hai He River, which flow into the estuary of Jiaozhou Bay (see Figure 1) and were obtained from the Jiaozhou Bay National Marine Ecosystem Research Station. In addition, nutrient data were collected at sampling points. The results revealed that the N, P, and silicate content at the river mouths (coastal areas of Jiaozhou Bay) were significantly higher than those in the interior of the bay. The occurrence of algal blooms is closely related to nutrient levels, and the large amounts of nutrients brought by rivers promote the development of nearshore phytoplankton in Jiaozhou Bay, leading to frequent algal blooms in this region. From 2000 to 2016, the outbreak of algal blooms in Jiaozhou Bay’s inner sea was not severe. However, since 2017, the outbreak of algal blooms in Jiaozhou Bay’s inland area has become more severe and did not abate until 2021.

### Reduction of false positives

After determining the optimal threshold for the Sentinel-2 dataset, we found that the optimal threshold for dataset 1C was larger than that for dataset 2A, and either 1C or 2A could extract the precise area. However, when both 1C and 2A used their optimal thresholds, their results were affected by false positives. As our analysis shows, applying the threshold for dataset 2A to 1C significantly reduced the number of false positives and the resulting algal bloom area.

We determined that the optimal threshold for detecting the presence of a harmful algal bloom using Sentinel-2 satellite level 2A data was 0.045. To compare and analyze the differences in false positives between level-1C and level-2A satellite extractions, we selected the months of January, February, March, October, November, and December, which rarely experience algal bloom outbreaks. Our goal was to extract data from datasets 1C and 2A with a threshold greater than 0.045 and then compare false positive rates during months when algal bloom outbreaks are uncommon.

The maximum area extracted from the level 2A data was only 31 $km^2$, and the mean value was 5.6 $km^2$. These results significantly affected our analysis of the algal outbreak area. Therefore, we chose to focus on the 1C data, which had a mean area value that was 93% less than that of dataset 2A. This significantly reduced the impact of false positives and made it easier to differentiate between real algal bloom outbreaks and false positives.

To further enhance our analysis, we decided to focus on a small rectangular area within Jiaozhou Bay. This allowed us to avoid confusion caused by false-positive results along the coast. In this small area, our analysis of level 1C data revealed a mean value that was 37.8% lower than level 2A data. This helped us discount false positives and obtain a more precise observation of the extent of the algal bloom outbreak.

**Figure 10:**
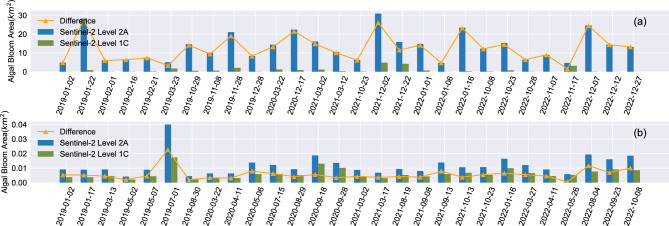
Comparison of the loss of algal bloom area and false-positive reduction. (**a**) shows the histogram of false positive reduction, (**b**) shows the histogram of the loss of algal blooms area.

In order to reduce false positives, The optimal level 2A threshold was utilized to extract level 1C data, and statistical analysis was performed (As shown in Figure 10). While the number of false positives decreased substantially, the area of algal blooms also decreased noticeably. Due to the presence of false positives, it was difficult to utilize level 2A data to evaluate the algal blooms. In contrast, the level 1C data eliminated the majority of false positives, making it simpler to observe the trend of the outbreak of algal blooms.

### Correlation analysis

#### Meteorological factors

**Figure 11:**
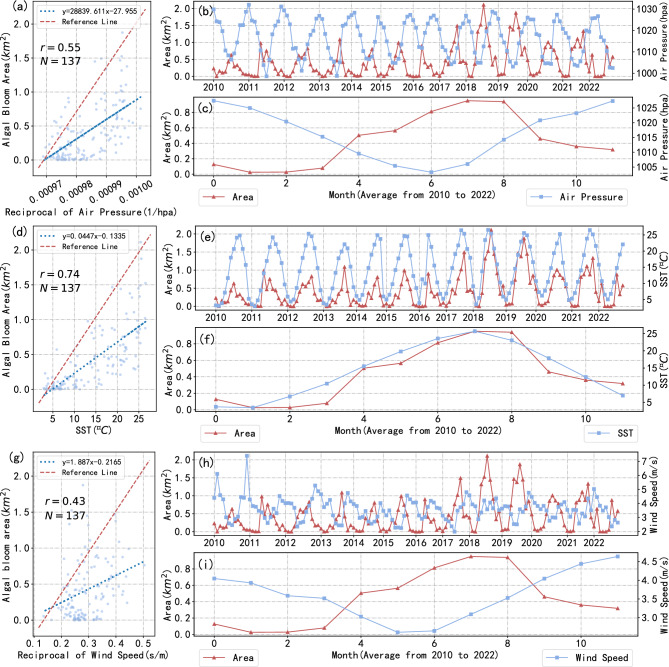
Analysis of the relationship between environmental factors and algal bloom outbreaks. (**a**, **d**, **g**) show scatter plots between the algal bloom area and air pressure(countdown), sea surface temperature(SST), and wind speed (countdown), respectively, and (**b**, **e**, **h**) show monthly average series of the algal bloom area and the air pressure, SST and the wind speed for November 2010–2022, and d (**c**, **f**, **i**) show the results of taking the average of the corresponding monthly values from 2010 to 2022.

Using MODIS data, we calculated monthly averages of algal bloom area and analyzed meteorological data to determine the factors contributing to algal bloom outbreaks in Jiaozhou Bay. We examined the available data to ascertain the relationship between these variables and algal bloom area. Our results show a weak negative correlation between wind speed and algal bloom area, consistent with previous findings (Figure 11)^38^. Additionally, our analysis reveals a significant inverse relationship between air pressure and algal bloom outbreaks, as well as a significant positive relationship between sea surface temperature (SST) and algal bloom outbreaks. We calculated the correlation coefficients between environmental factors and algal bloom outbreaks (Table 1). These findings suggest that algal bloom outbreaks are more likely to occur when wind speed is low, air pressure is low, and temperature is high. With these environmental factors in mind, appropriate measures can be taken to control algal bloom outbreaks. Our analysis of the spatial distribution of algal bloom area in Jiaozhou Bay indicates that outbreaks are more severe in the bay’s coastal regions. The area of algal blooms decreases progressively from the coast to the inner waters, with the majority of outbreak areas concentrated on the coast, middle, and estuary. From 2000 to 2016, algal bloom outbreaks in Jiaozhou Bay’s inner waters were not severe. However, beginning in 2017, outbreaks in the bay’s inner waters grew steadily worse until 2021, when they were ultimately mitigated.

**Table 1:** Correlation coefficients between the algal bloom area and three meteorological factors: air pressure, wind speed and SST.

	Air pressure	SST	Wind speed
Pearson’s $r^2$	0.30	0.55	0.18
$\rho$^1^	−0.58	0.8	−0.32

$^{1}$Here $\rho$ represents the Spearman’s rank correlation coefficient.

#### Seawater quality factors

**Figure 12:**
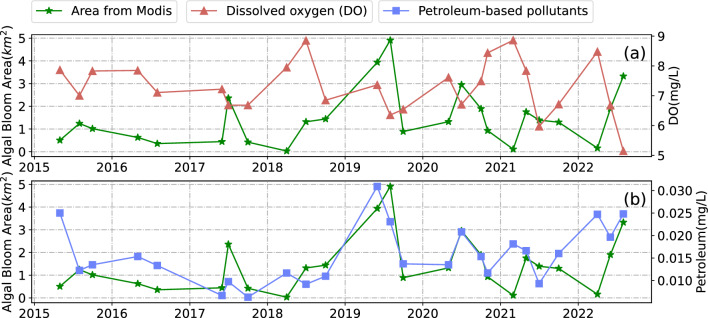
Time series of petroleum pollutants, DO and algal bloom area extracted from MODIS.

**Figure 13:**
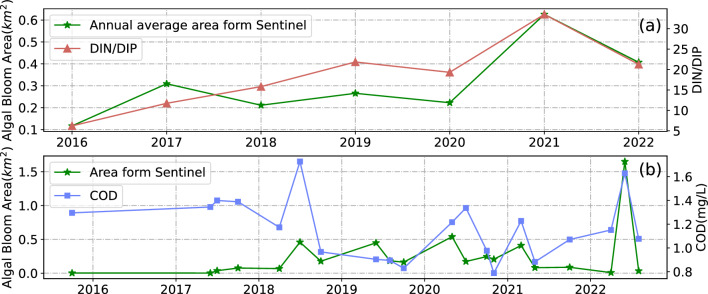
Yearly time series of DIN/DIP, COD and algal bloom area extracted from Sentinel-2 data.

In this study, seawater quality data collected in Jiaozhou Bay between 2014 and 2022 was used to investigate the relationship between water quality and algal bloom outbreaks in conjunction with algal bloom area extracted from MODIS and Sentinel-2 data. MODIS satellite images within 5 to 7 days before and after water quality sampling were chosen for extracting algal bloom areas, and the extracted areas were then averaged for comparison analysis. As Fig. 12 shows, there was a clear correlation between algal bloom area and petroleum pollutants, which could be attributed to the fact that petroleum hydrocarbons nourish algal organisms. In contrast, there was an inverse relationship between algal bloom area and dissolved oxygen (DO), which may result from oxygen depletion in the water column caused by algal growth. The previously described approach was not suitable for Sentinel-2 satellite data due to its long sampling interval. Consequently, monthly and annual averages of algal bloom area derived from Sentinel-2 satellite data were used to analyze the correlation with corresponding water quality data. The results (Fig. 13) indicate a certain correlation between algal bloom area, DIN/DIP, and COD concentrations. The results of their regression analysis are presented in Table 2.

### Predicted results

**Figure 14:**
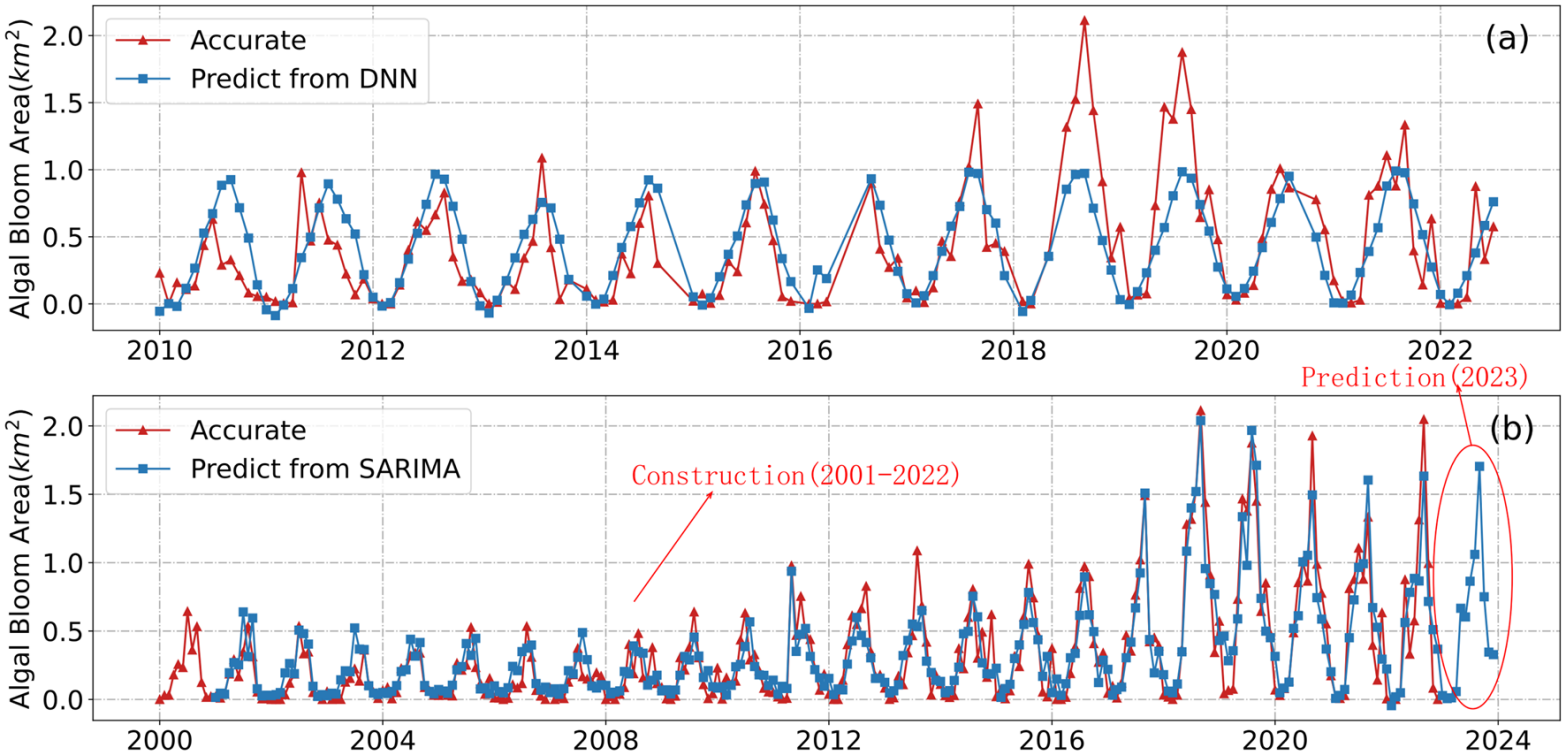
Prediction of algal bloom area by DNN (**a**) and SARIMA (**b**).

Figure 14a The results from the DNN neural network model closely agree with the actual values, indicating the model’s robustness and consistent trend. Due to the unavailability of meteorological data during certain periods, some monthly area data could not be used in the forecast. However, since the prediction is based solely on meteorological factors, it is impossible to accurately foresee abrupt increases in algal bloom area due to other factors. By integrating seawater quality composition, the model’s accuracy can be improved. After optimizing the model’s hyperparameters, its performance was assessed and summarized (Table 3). The final test set has an $R^2$ value of 0.65, indicating acceptable prediction accuracy.

**Table 2:** Regression results.

Significant indicators	Coefficient	Std. err.	t	P>|t|
DIN/DIP	0.02	0.006	2.88	0.028
SST	0.06	0.005	12.47	0
Petroleum	97.9	30.7	3.18	0.005
DO	−0.71	0.21	−3.33	0.004

**Table 3:** DNN model evaluation.

	MSE	RMSE	MAE	MAPE	$R^2$
Training sets	0.097	0.311	0.219	66.763	0.541
Cross-validation sets	0.105	0.314	0.231	151.714	0.467
Test sets	0.055	0.234	0.183	65.706	0.651

According to the predicted results of the SARIMA model (Fig. 14b), algal bloom outbreaks are highly seasonal. This is due to cyclical temperature changes accompanying the seasons and the disruption of the nitrogen-phosphorus ratio balance in the water column during the summer fishing season peak. Since algal blooms have a strong correlation with temperature, nitrogen, and phosphorus ratios, this leads to seasonal outbreaks. An upward trend in algal bloom outbreaks is observed, with substantially stronger outbreaks in the second and third quarters than in the first and fourth quarters. We used an additive model of seasonal decomposition to quantify the impact of seasonal factors on algal bloom outbreaks.

**Table 4:** Seasonal factors for the variation of algal bloom area.

Month	1	2	3	4	5	6	7	8	9	10	11	12
SF$^*$	−0.22	−0.29	−0.30	−0.26	0.03	0.12	0.30	0.39	0.40	0.03	−0.09	−0.11

$^*$The seasonal factor.

According to the results shown in Table 4, seasonal factors are positive for May to October and negative for January to April and November to December. This suggests that algal bloom outbreaks are more severe in the second and third quarters than in the first and fourth. The most intense outbreak occurred in September, with an area larger than the annual average of 0.398 $km^2$, while the smallest occurred in March, with an area smaller than the annual average of 0.298 $km^2$. We removed outliers from two decades of historical algal bloom outbreak data before iteratively estimating parameters to obtain an iterative expression for the time series (2). All significance levels are less than 5% based on the estimation results (Table 5), indicating a good fit.

3 $$\begin{aligned} y_{t}=0.015+y_{t-12}+\varepsilon _{t}-0.217 \varepsilon _{t-1}+0.689 \varepsilon _{t-12}-0.150 \varepsilon _{t-13} \end{aligned}$$

where $y_t$ and $y_{t-12}$ are the actual observed area of algal blooms in periods t and t-12, respectively. $\varepsilon _{i}$ is the white noise series with variance $\sigma ^{2}$, which represents the difference between the predicted value and the observed value in period i. In this equation i takes t,t-1,t-12,t-13.

**Table 5:** Evaluation of estimation results.

Parameter	Estimation result	Standard error	t	Significance
$\alpha _0$ *	0.015	0.004	3.28	0.001
$\theta$ *	−0.22	0.06	−3.46	0.001
$\Theta$ *	0.69	0.05	12.66	0

$^*$ $\alpha _0$,$\theta$,$\Theta$ are estimates of the parameters to be estimated in Eq. (2)

## Discussion

**Figure 15:**
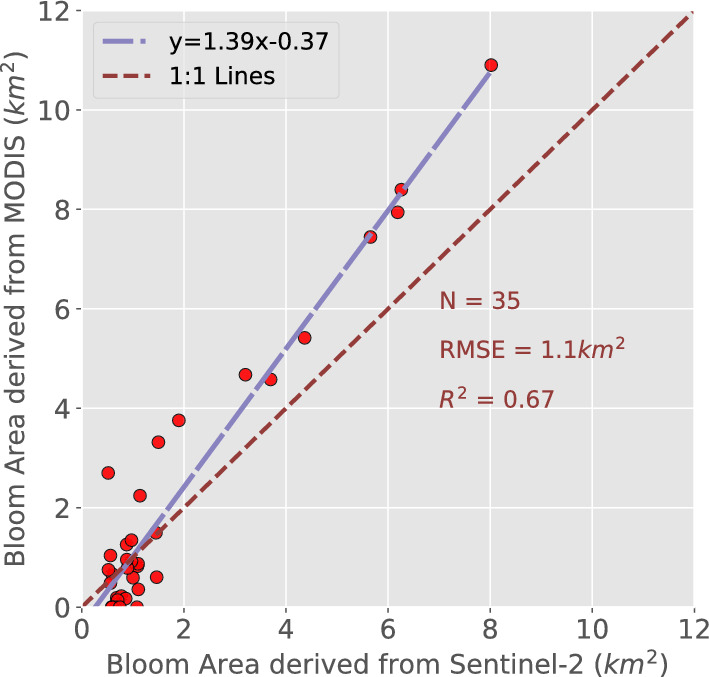
Comparison of algal bloom area derived from MODIS and Sentinel-2 data.

For comparative analysis, we obtained MODIS datasets corresponding to the Sentinel-2 data. To reduce the impact of false positives during extraction, we excluded data with minor values based on predetermined thresholds and retained only data collected during algal bloom periods. The data was then filtered to yield 35 corresponding data points. After analyzing the correlation between the two datasets using scatter plots, we calculated an $R^2$ value of 0.67 (Fig. 15). Due to the 500-meter resolution of the MODIS satellite’s observational pixel, the vast majority of algal blooms could be included, even at the boundary. However, despite our best efforts to modify the threshold value, non-algal material may still be included, resulting in a high algal bloom area. Although the Red(620–670nm) and Green(545–565nm) bands of MODIS have some degree of accuracy, NIR(841–876nm) and Blue(459–479nm) bands have a high degree of uncertainty and may not be suitable for monitoring algal blooms at a large scale^39^. The effectiveness of Sentinel-2 data with a 10-meter resolution in extracting algal bloom areas is unstable due to the influence of clouds and the sampling period. Using the FAI threshold extraction method, the algal bloom region can be extracted from certain images with relatively high precision. However, its long resampling interval makes it unsuitable for analyzing changes in algal bloom area. Additionally, its higher resolution makes it susceptible to the influence of coastal infrastructure on ocean color, resulting in erroneous estimates of the extracted algal bloom area^40^.

In this study, algal bloom data extracted from the MODIS dataset were analyzed using the average of observed non-zero data within a range of 5 to 7 days before and after the water quality observation time. This approach was necessary due to the temporal dispersion of water quality measurements, which were conducted three to four times per year. Using monthly averages of algal bloom area and water quality data may introduce errors due to their temporal inconsistency. Although it would be ideal to match the precise date of water quality data collection with the one-day resampling nature of the MODIS satellite, de-cloud processing makes it impossible to precisely align the satellite observation period with water quality monitoring. Therefore, the average value of observed water bloom area within 5–7 days of water quality monitoring was used as corresponding data. The 5-day resampling time for Sentinel-2 satellite data highlights discontinuity following de-clouding processing, necessitating the use of monthly averaged water bloom area values for analysis, which may introduce some error. Additionally, collecting water quality data before and after algal blooms for comparative analysis would enable more precise investigation of their causes. The inconsistency between satellite sampling and water quality monitoring can result in a lack of correlation and analytical error.

Algal blooms result from a combination of physiological mechanisms and environmental factors, including water temperature, wind speed, atmospheric pressure, and water quality. Nitrogen and phosphorus have been identified as significant contributors to algal bloom outbreaks^41^. Research indicates that silicate and phosphorus are the primary limiting factors for planktonic algae expansion in the Jiaozhou Bay region^42^. The majority of Jiaozhou Bay’s total DIN and DIP discharges come from land-based sources, accounting for 93% and 98%, respectively^43, 44^. Land-based pollutants enter Jiaozhou Bay via inlet rivers, most of which are currently flow-interrupted. Four tributary rivers, the Licun, Dagu, Ink, and Haibe Rivers, were analyzed in 2014 and 2015 when they had water flow. Nutrient ratios were determined by analyzing total nitrogen, total phosphorus, and silicate content at the rivers’ entrances to Jiaozhou Bay. The N/P ratio was 25.8±17.1, the N/Si ratio was 3.35±1.35, and the Si/P ratio was 8.9±6.4. N/Si values tend to remain stable in Jiaozhou Bay, while the N/P ratio fluctuates the most. Furthermore, P has a greater impact on planktonic algae growth than Si in Jiaozhou Bay. Some studies have indicated that Jiaozhou Bay has become a “phosphorus-limited” environment , consistent with the findings of this study^45, 46^.

Researchers investigating 17 lakes worldwide have shown that lower N/P ratios promote algal growth^47^, but some studies have also shown that algal outbreaks occur when the N/P ratio is high, suggesting that a low N/P ratio is a result rather than a cause of the outbreak^48^. It should be noted that the N and P requirements of algae vary depending on the environment and species. This study’s analysis concluded that there is a positive correlation between the N/P ratio and algal bloom area. The results also demonstrated that the N/Si value tends to be stable, indicating that algae in Jiaozhou Bay are highly sensitive to changes in phosphorus levels. The highest observed DIN/DIP value reached 387, and the N/P ratio at several observation sites exceeded 100 in summer, surpassing the Redfield ratio^49^. However, the ratio decreased in winter. For example, during a severe algal bloom in 2019, the average N/P ratio in August was 62, while in October it was 14.9, approaching the Redfield ratio. These findings suggest that sudden increases in phosphorus content due to external factors could trigger large algal bloom outbreaks, leading to a decrease in the nitrogen-phosphorus ratio.

In this study, a positive correlation was observed between algal bloom area and COD content. This can be attributed to the release of organic matter by algae during their growth cycle, particularly during normal growth and metabolism stages when extracellular organic matter (EOM) is released into the water column, leading to a significant increase in COD content. Other research^50^ confirms this idea, indicating that an abrupt spike in COD concentration results from an algal bloom epidemic . However, due to the influence of the long resampling interval for Sentinel-2 observation and nutrient concentration measurement, monthly average data was used for analysis, and thus the lag in COD content was not very clear. Nevertheless, the study still demonstrates a correlation between COD and algal bloom area.

## Conclusion

Examining the long-term time series of algal blooms is essential to comprehend the ecological changes in Jiaozhou Bay and mitigate the risks posed by algal blooms. Using MODIS and Sentinel-2 satellite datasets, this study observed the spatial and temporal variations of algal blooms from 2000 to 2022, analyzed the factors driving the outbreak of algal blooms in the Jiaozhou Bay region, and modeled the changes of algal blooms in the region using deep learning and predicted that with SARIMA models.

The study found that the incidence of algal blooms in Jiaozhou Bay was comparatively stable from 2000 to 2016, but has increased since 2017, especially during the summer, indicating a more pronounced seasonality. The algal bloom outbreak areas were primarily concentrated along the coast, in the midsection, and at the estuary of the bay. Meteorological factors such as sea surface temperature, wind speed, and air pressure, as well as water quality factors such as dissolved oxygen, nitrogen to phosphorus ratio, chemical oxygen demand, and petroleum pollutants, were identified as important drivers of algal bloom outbreaks.

In addition, the study revealed that the algal bloom area computed using MODIS satellites was generally larger than those calculated using Sentinel-2 satellites, which was a result of the sensor characteristics of the satellites. Finally, time series regression based on deep learning and predictions based on SARIMA models were developed to reproduce and forecast variations in algal bloom area at a monthly scale.

In conclusion, this study provides critical data support for the study of algae blooms in the Jiaozhou Bay region, offers reference advice to governmental departments for emergency measures to manage algal blooms in the area, and offers valuable insights for future research on algal bloom outbreaks in bays around the world.

### Supplementary Information

Supplementary Information.

## Data Availability

The water quality monitoring datasets of Jiaozhou Bay used during the current study are not publicly available due to the provision of the data provider (Jiaozhou Bay Marine Ecosystem Research Station, Chinese Ecosystem Research Network), but are available from the corresponding author on reasonable request.
