# Supplementary material for: Comprehensive study of algal blooms variation in Jiaozhou Bay based on google earth engine and deep learning
Source: Sci Rep. 2023 Aug 25;13:13930. doi: 10.1038/s41598-023-41138-w (PMC10457358; doi:10.1038/s41598-023-41138-w)
Supplement: Supplementary file 1 — Supplementary Information. [file 41598_2023_41138_MOESM1_ESM.pdf]

Supplementary Material

We present the results of a quantitative discussion of multiple regression and time series models. The relevant data and results used to draw conclusions are provided in this appendix.

S1 Regression analysis

Prior to the regression analysis, the lasso regression is employed to screen the variables, and the parameter estimate can be expressed as:

$$\hat{\beta} = arg\min_{\hat{\beta}} \left[\sum_{i=1}^{n} \left(y_i - x_i{'} \hat{\beta} \right)^2 + \lambda \sum_{i=1}^{k} \left| \hat{\beta}_i \right| \right] \quad (1)$$

Following data normalization, a 10-fold cross-validation method is utilized to select the smallest MSPE (mean square prediction error) to adjust the parameters. The optimal results obtained by setting the random number seed to 520 are presented in Table 1 and Table 2.

Table S1 Estimated value of λ with minimum mean square prediction error

λ	**MSPE**	**St.dev.**
8.6244741	1.0479	0.7753

Table S2 Variables screened out by lasso regression and their parameter estimation results

**Filtered variables**	**Lasso regression Estimation Parameters**	**OLS regression estimation parameters**
N/P	0.1855	0.7618
Cons.	0.0000	0.0000

Afterwards, we conducted multiple linear regression analysis on the nitrogen-to-phosphorus ratio and the algal bloom area, and constructed the following model:

$$BLOOMS_i = \alpha + \beta_1 NP_i + \varepsilon_i \quad (2)$$

where $BLOOMS_i$ represents the algal bloom area in the i year in the sample, and NP_i represents the nitrogen-to-phosphorus ratio in the i year， α and β represent the regression coefficients and ε_i denotes a perturbation term that is unobservable and satisfies certain conditions.

Before getting the regression results, it is necessary to test the heteroscedasticity first. Observing the residual graph (Figure S1), it can be seen that the residuals are distributed in an S-shape on both sides of the Y=0 line, and the data has variance inhomogeneity.

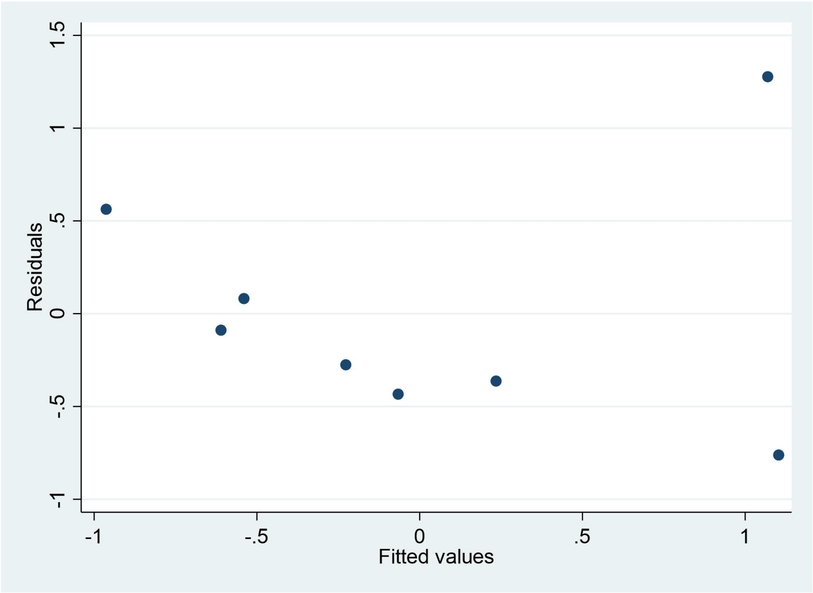

Figure S1 The residual plot of the first set of data

At the same time, after BP test and White test, respectively set the confidence level of 95% and 90%, it is found that heteroscedasticity does exist.

Table S3 Heteroscedasticity Test Results of the first set of data

Testing method	Null hypothesis	result
BP test	no heteroscedasticity	Prob > chi2 = 0.0437
White test	no heteroscedasticity	Prob > chi2 = 0.0627

Therefore, the OLS + robust standard error processing method is used here to eliminate the influence of heteroscedasticity, and through the joint significance test, the following final regression results are obtained:

Table S4 Regression result of the first set of data

	Coefficient	Std. err.	t	P>t	95% confidence interval	
N/P	0.0166	0.0057	2.8800	0.0280	-0.2899	0.0283
Cons.	0.1204	0.1209	1.0000	0.3580	-0.4162	0.1754

Similar to the above process, for another set of data, the monthly average data of MODIS and environmental factors can be regressed to obtain the following significant factors, and the following results can be obtained:

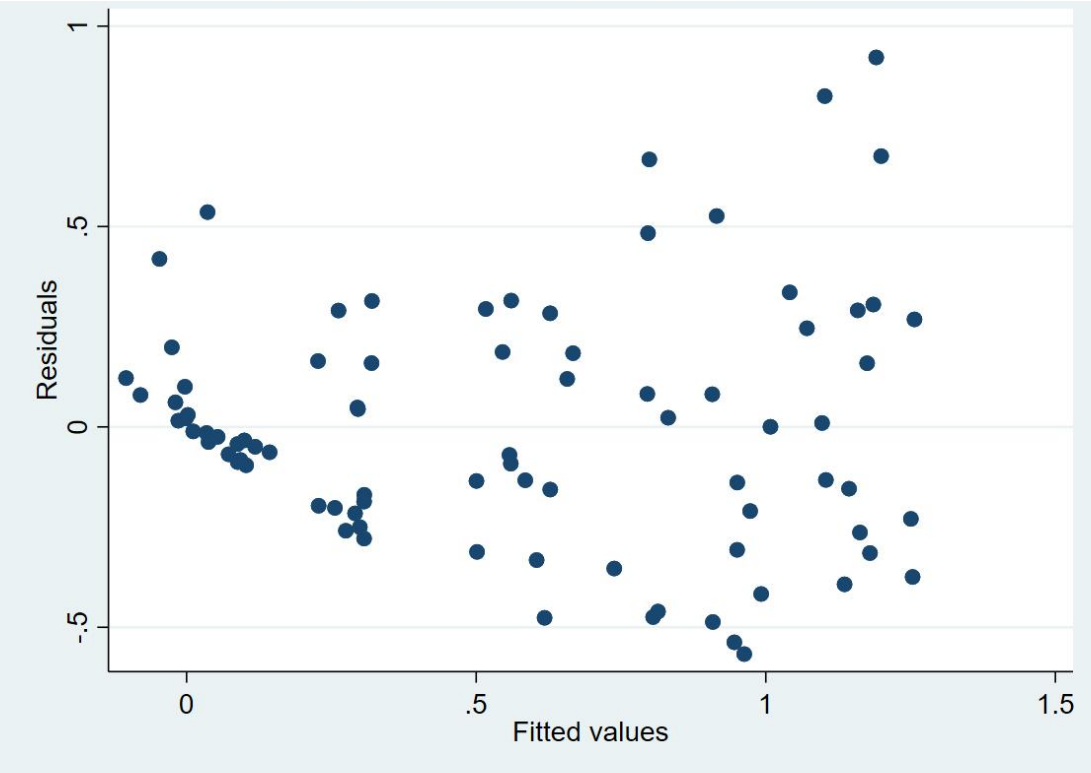

Figure S2 The residual plot of the second set of data

Table S5 Regression result of the second set of data

	Coefficient	Std. err.	t	P>t	95% confidence interval	
SST	0.0612	0.0049	12.4700	0.0000	0.0507	0.0716
Cons.	0.5775	0.2275	-2.5400	0.0130	-1.0391	1.1584

Using Sentinel data to regress the chemical composition extracted from the ocean, the following results can be obtained:

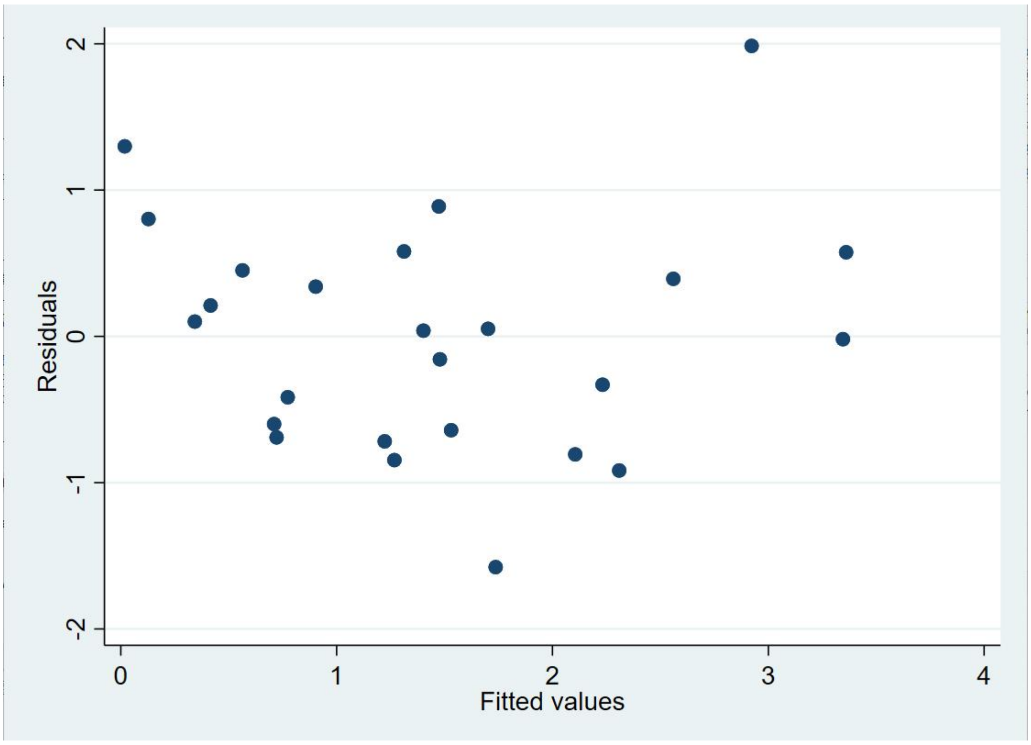

Figure S3 The residual plot of the third set of data

Table S6 Regression result of the third set of data

	Coefficient	Std. err.	t	P>t	95% confidence interval	
Petroleum (mgL)	97.9000	30.7000	3.1800	0.0050	33.5000	162.0000
Dissolved oxygen (mgL)	-0.7065	0.2121	3.3300	0.0040	-1.1504	0.2626
Cons.	33.5000	23.6000	1.4200	0.17300	16.0000	83.0000

Based on the standardized regression, we used Stata software to calculate the standardized regression coefficients to determine the degree of importance of each covariate, and the results are shown in the table below. We judged the degree of importance by the size of the absolute value of Beta, and the larger the value, the more significant the influence of the indicator on the algal bloom outbreak.

Table S7 Standardized regression results

**Variables**	**Coefficient**	**Beta**	**Variables**	**Coefficient**	**Beta**
Temperature	-0.00946	-0.1369	PH value	-3.04318	-0.1723
Wind Speed	0.08729	0.1089	Active phosphate	-37.9456	-0.2967
Air pressure	-0.00416	-0.0662	COD	0.07591	0.01495
Sea temperature	0.06799	0.9385	TN	2.0405	0.19827
Dissolved oxygen	-0.70397	-0.5124	Petroleum	100.3026	0.5151

S2 Time Series Forecasting

For further analysis, we utilized an additive model of seasonal decomposition to quantify the impact of seasonality on algal blooms. The figure below illustrates the results of the seasonal decomposition.

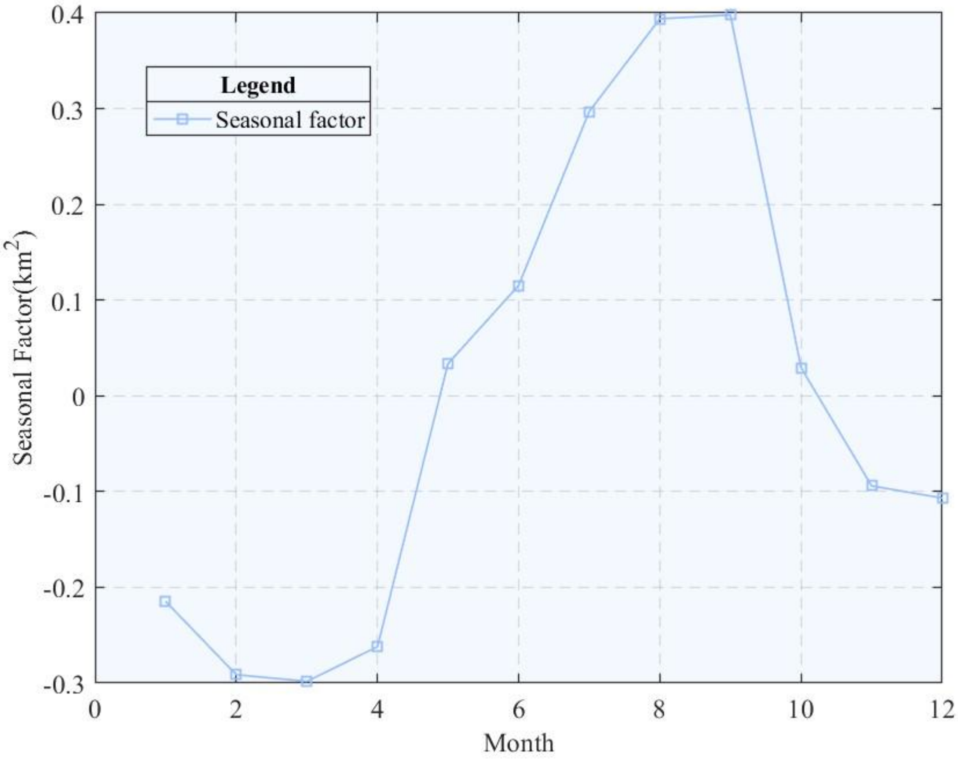

Figure S4 Seasonal factor of algal bloom area.

It is evident that the seasonal factors from May to October are positive, while the seasonal factors from January to April and November to December are negative. This suggests that algal blooms are more severe in the second and third quarters compared to the first and fourth quarters. Moreover, the most severe outbreak occurred in September, with an area higher than the annual average of 0.397,9 square kilometers while the lowest outbreak occurred in March, with an area lower than the annual average of 0.298,2 square kilometers.

Furthermore, the four components obtained by time series decomposition are presented in the figure below. These components are more regular than the original series, which facilitates the fitting and prediction of algal blooms.

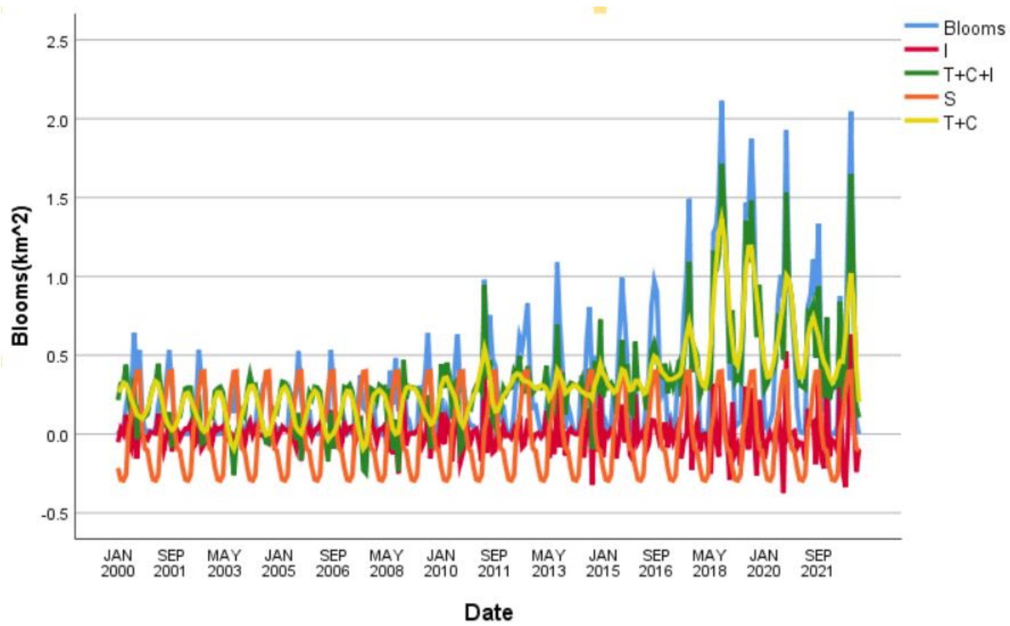

Figure S5 Time Series Decomposition Chart. The figure above displays the four modules obtained from the time series decomposition, which are more regular than the original series and can be used for predicting algal blooms. The pink line represents the irregular change curve, with a much smaller amplitude than the other curves. This indicates that the impact of unforeseen accidental factors on the time series is negligible and can be ignored in the analysis. This also confirms the feasibility of our time series analysis. The green line represents the seasonally adjusted sequence, which removes the seasonal effect and shows the overall trend of time-varying changes. It can be observed that there is a clear upward trend, indicating that the algal bloom in Jiaozhou Bay is becoming more severe. The orange line is the seasonal adjustment factor, which simply reflects the periodic changes in the value of algal blooms caused by seasonal variations. The fluctuations caused by seasonal changes are around 1 square kilometers, demonstrating that seasonal fluctuations have a significant impact on algal blooms. The yellow line represents the trend cycle component, which characterizes the long-term trend and cycle change and has a similar meaning to the seasonally adjusted sequence.

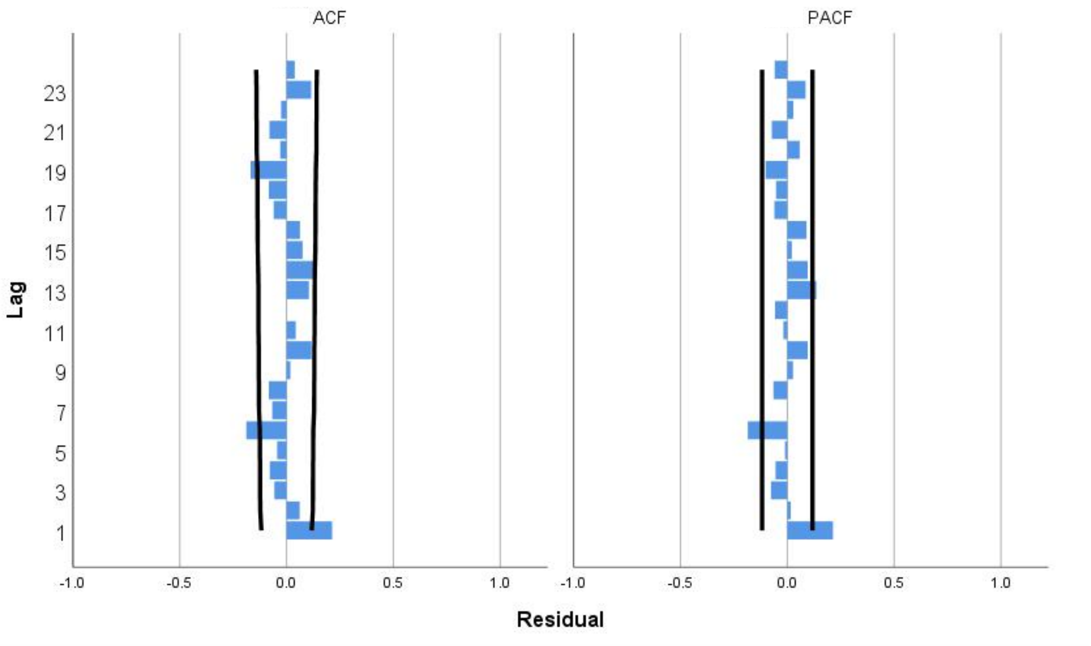

Figure S6 Residual Plot. The ACF and PACF graphs of the residuals indicate that the autocorrelation coefficients and partial autocorrelation coefficients of all lag orders are not significantly different from 0.

Moreover, the p-value in the table below is 0.305, which implies that we cannot reject the null hypothesis that the residual is a white noise sequence. Therefore, the SARIMA (0, 0, 1) (0, 1, 1) model can effectively identify the algal bloom data.

Table S8 White Noise Sequence Tests

		Model Fit Statistics	Young Box Q(18)			
Model	number of predictors	Stationary R-squared	statistics	DF	significant	number of outliers
Algal bloom area - model	0	0.560	18.321	16	.305	5

The model fitting degree table below shows a goodness of fit of 0.836, close to 1, indicating that the estimation effect of the model is highly accurate.

Table S9 The model fitting degree table

Fit Statistics	average value
Stationary R-squared	0.5600
R-squared	**0.8360**
RMSE	0.1670
MAPE	1759.1610
MaxAPE	196610.2120
MAE	0.1190

Based on these results, we can forecast the expected algal bloom area for the year 2023.

Table S10 Forecast result table

Month	Predictive value(km^2)
1	0.0280
2	0.0062
3	0.0117
4	0.0578
5	0.6666
6	0.6033
7	0.8643
8	1.0603
9	1.7042
10	0.7506
11	0.3460
12	0.3276
